# Arterial wave dynamics preservation upon orthostatic stress: a modelling perspective

**DOI:** 10.1098/rsos.221257

**Published:** 2023-03-01

**Authors:** Matteo Fois, Luca Ridolfi, Stefania Scarsoglio

**Affiliations:** ^1^ Department of Mechanical and Aerospace Engineering, Politecnico di Torino, Corso Duca degli Abruzzi 24, Turin 10129, Italy; ^2^ Department of Environmental, Land and Infrastructure Engineering, Politecnico di Torino, Corso Duca degli Abruzzi 24, Turin 10129, Italy

**Keywords:** arterial haemodynamics, orthostatic stress, gravity, cardiovascular modelling, pressure-flow wave analysis

## Abstract

Pressure-flow travelling waves are a key topic for understanding arterial haemodynamics. However, wave transmission and reflection processes induced by body posture changes have not been thoroughly explored yet. Current *in vivo* research has shown that the amount of wave reflection detected at a central level (ascending aorta, aortic arch) decreases during tilting to the upright position, despite the widely proved stiffening of the cardiovascular system. It is known that the arterial system is optimized when in the supine position, i.e. propagation of direct waves is enabled and reflected waves are trapped, protecting the heart; however, it is not known whether this is preserved with postural changes. To shed light on these aspects, we propose a multi-scale modelling approach to inquire into posture-induced arterial wave dynamics elicited by simulated head-up tilting. In spite of remarkable adaptation of the human vasculature following posture changes, our analysis shows that, upon tilting from supine to upright: (i) vessel lumens at arterial bifurcations remain well matched in the forward direction, (ii) wave reflection at central level is reduced due to the backward propagation of weakened pressure waves produced by cerebral autoregulation, and (iii) backward wave trapping is preserved.

## 1. Introduction

The human cardiovascular system (CVS) undergoes severe haemodynamic alterations when experiencing orthostatic stress [1,2], that is when a subject either stands up, sits or is tilted head-up from supine on a rotating table. Among the most widely observed responses, clinical trials have shown accelerated heart rhythm and reduced circulating blood volume (cardiac output), together with marked anatomical changes of human vessels including augmented peripheral resistance and diminished venous tone, leading to an overall increase of arterial wave speed and stiffening of the systemic vasculature [3–5].

In spite of the fact that the dynamics of pressure and flow waves travelling throughout the arterial tree are crucial to understand, monitor and evaluate CVS functioning [6–11], their dependence on the body posture remains unclear. The importance of wave patterns lies in the fact that they reflect the mechanical and geometric characteristics of the arterial system. Indeed, arterial pressure and flow waves originate at the aortic root level due to contraction and relaxation of the left ventricle. They enter the arterial tree by the aortic valve and travel down the aorta through successive bifurcations, reaching all arterial peripheral branches. Meanwhile, they are continuously reflected giving rise to backward propagating waves [7,8,12,13]. Therefore, at any point throughout the arterial tree, the local pressure and flow waves are the result of the superposition of forward and backward travelling waves.

Interestingly, in the supine position the CVS is optimized from the point of view of waves transmission and reflection phenomena. In fact, direct (i.e. forward) waves are transmitted with low losses through arterial bifurcations [13,14] (due to the optimal cross-section area matching between parent and daughter vessels) and across tapered vessels. Differently, reflected waves—particularly those produced at terminal branches—are trapped and cannot travel back along the arterial tree reaching the aortic valve, resulting in the advantageous ‘wave trapping’ effect documented by many researchers [14–16]. This configuration represents a natural protection of the heart from reflected waves, meanwhile reducing cardiac workload due to the enhanced forward waves propagation. Nevertheless, despite the fact that most of human life takes place in a standing posture, little is known concerning wave transmission and reflection phenomena on a standing subject. In particular, no investigation has been conducted on whether and how this favourable CVS configuration encountered at supine position is maintained or lost with body posture changing to upright standing.

Some effort has been made to obtain *in vivo* measurements of arterial wave reflection upon varying posture [17–20]. These studies highlighted that central (i.e. ascending aorta, aortic arch) wave reflection seems to be reduced with tilting to the upright posture, despite the well-proved overall CVS stiffening driven by augmented peripheral resistance and reduced vessels tone and compliance (mainly venous), as mirrored by the globally increased wave velocity [13,21,22]. Therefore, such results remain contradictory, deserving better elucidation.

We aim to fill these gaps by exploring the posture-induced wave dynamics elicited under orthostatic stress. To this end, we propose a numerical investigation of the arterial wave dynamics upon simulated head-up tilt from supine to upright standing posture. Modelling tools have never been employed so far to dig into this topic, although multi-scale modelling of blood motion and pulse wave dynamics through large vessels are gathering increasing popularity in the fluid dynamics community [23–25] due to their high versatility and usefulness. In our analysis, we adopted a multi-scale and closed-loop model of the entire human circulation developed and validated under different working conditions [26–28]. The model is composed of a one-dimensional distributed network of the arterial tree, connected to a zero-dimensional lumped parametrization of the peripheral microcirculation, venous return and cardiopulmonary circulation. The model accounts for short-term regulation of central arterial and venous pressure, as well as for the autoregulation of cerebral blood flow.

Using the mathematical model, we carried out wave analysis (WA) focusing on the role of body posture. Wave reflections originating from all parts of the arterial network were quantified at supine and standing postures accounting for: (i) reflections at arterial bifurcations [13,14,29] both in the forward (parent–daughters) and backward (daughters–parent) directions, (ii) tapering of arterial vessels [29–31], and (iii) reflection produced at peripheral terminal branches [6,13,29]. We also performed wave intensity analysis (WIA) [8,12,29,32] along the aorta to highlight interesting wave patterns and track backward travelling waves from their peripheral originating point up to each investigation site. Finally, we assessed backward wave trapping [14–16] along the aorta and its behaviour in response to body posture variation.

## 2. Methodology

We describe here the key elements of the CVS model adopted in the present study. Further details, equations, model settings and WA/WIA metrics derivation are given in the electronic supplementary material and in our previous works [26–28,33].

### 2.1. The cardiovascular model

**Figure 1.**
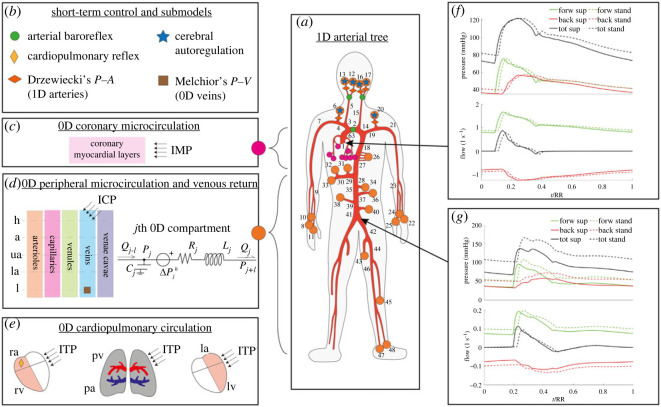
Schematic illustration of the CVS model. (*a*) The one-dimensional arterial tree; black numbers identify one-dimensional arterial vessels; orange and purple circles at one-dimensional terminal branches refer to the connections with the zero-dimensional arteriolar (*d*) and coronary microvascular (*c*) compartments, where IMP is the intramyocardial pressure. (*d*) The zero-dimensional peripheral and venous return compartments (h: head, a: arms, ua: upper abdomen, la: lower abdomen, l: legs) as well as a sketch of the *j*-th compartment electric analogue, where *j* − 1 is the preceding compartment, *j* + 1 is the following compartment, *Q* is the blood flow rate, *P* is the blood pressure, Δ*P*^*h*^ is the hydrostatic pressure contribution, ICP is the intracranial pressure and *R*, *L* and *C* are the lumped resistance, inertance and compliance, respectively. (*e*) The cardiopulmonary circulation, where ra, rv, la and lv are right atrium and ventricle, left atrium and ventricle, respectively, pv and pa are pulmonary veins and arteries, and ITP is the intrathoracic pressure. (*b*) The different submodels plugged to the CVS model (*V* is the blood volume, *A* is the vessel cross-section area). For Drzewiecki’s and Melchior’s submodels please refer to Drzewiecki *et al.* [34] and Melchior *et al.* [35], respectively. (*f*,*g*) Two examples of blood pressure and flow waveforms (tot: total, forw: forward, back: backward signals) taken at two arterial sites (aortic root no. 1 and iliac bifurcation no. 41) for the supine (sup, solid lines) and the standing (stand, dashed lines) cases, respectively. Time *t* is normalized with the heartbeat duration, RR.

The model is multi-scale and closed-loop, encompassing a one-dimensional description of the arterial tree and a zero-dimensional lumped parametrization of the peripheral microcirculation, the venous return and the cardiopulmonary circulation, as illustrated in figure 1.

Blood motion through one-dimensional systemic arteries (figure 1*a*) is governed by the axisymmetric form of the Navier–Stokes equations for mass and momentum balance (equations (S1) and (S2) in the electronic supplementary material), where gravity influence is accounted for through Stevino’s law considering the orientation of the vessel with respect to the body axis and the horizontal reference. A constitutive tube-law accounting for the visco-elastic behaviour of arterial walls is adopted to represent blood pressure-deformation of arterial vessels (electronic supplementary material, equation (S3)).

The zero-dimensional side of the CVS model includes lumped arteriolar, capillary, venular and venous resistance, inertance and compliance (RLC) compartments organized into five body regions, i.e. head, arms, upper and lower abdomen and legs (figure 1*d*); in addition, two inferior and one superior venae cavae compartments are enclosed (electronic supplementary material, equations (S4)–(S6)). The cardiopulmonary circulation includes zero-dimensional time-varying elastance models for the four cardiac chambers (electronic supplementary material, equation (S7)), non-ideal diodes models of the heart valves, plus an arterial and a venous RC pulmonary compartment.

The CVS model accounts for the action of specific extravascular pressures onto given vascular districts: the intramyocardial pressure (IMP [28], figure 1*c*), the intrathoracic pressure (ITP [27], figure 1*e*, electronic supplementary material, equation (S8)) and the intracranial pressure (ICP [26], figure 1*d*). ITP and ICP depend on the body posture [26,27].

The model is equipped with short-term regulation mechanisms (indicated in figure 1*a*,*b*) for the maintenance of the system homeostasis. Such short-term regulations include a baroreflex and a cardiopulmonary reflex model (electronic supplementary material, equation (S9)) controlling the heart rate, cardiac contractility, peripheral resistance of arteriolar and capillary compartments as well as venous and venular tone (volumes and compliances). A cerebral autoregulation model is also embedded to control cerebral arteriolar compliance and resistance in order to preserve nearly constant values of cerebral blood flow.

### 2.2. Wave separation and wave analysis

Considering local pressure *P* and flow *Q* signals at a given point of the arterial network, their forward (*P*_*f*_ and *Q*_*f*_, respectively) and backward (*P*_*b*_, *Q*_*b*_) travelling components are expressed as in [13,15,29],

2.1 $$P_{\;f,b}=\frac{P\pm Z_{c}Q}{2}$$

and

2.2 $$Q_{\;f,b}=\frac{Q\pm P/Z_{c}}{2},$$

where the signs ‘+’ and ‘−’ refer to forward and backward waves, respectively, while *Z*_*c*_ is the local vessel characteristic impedance. Examples of pressure and flow wave separation performed at two points of the arterial tree are displayed in figure 1*f*,*g*.

**Figure 2.**
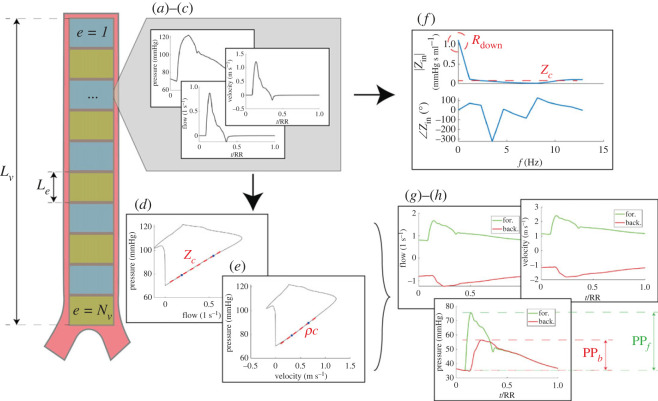
Wave analysis operational flow (examples refer to the aortic root vessel no. 1). For a given one-dimensional arterial vessel *v* of length *L*_*v*_, $e=1,\ldots ,N_{v}$ are its discretized segments of length *L*_*e*_. For each single segment *e*, (*a*–*c*) displays the local pressure, flow and velocity time signals. (*d*,*e*) The *PQ*- and *PU*-loops, respectively (*ρ* is blood density, *c* is the local wave speed and *Z*_*c*_ is the local vessel characteristic impedance). (*g*,*h*) The separated forward (forw, green) and backward (back, red) components of pressure, flow and velocity waveforms for the *e*th segments, with PP_*f*_ and PP_*b*_ being the pulse pressure of the forward and backward pressure signals, respectively (time *t* is normalized with the heartbeat duration, RR). (*f*) The frequency content of the input impedance *Z*_in_ in terms of its modulus |.| and phase ∠., resulting from Fourier decomposition of the pressure and flow signals associated with the *e*th segment (*R*_down_ is the downstream resistance with respect to the considered site, *Z*_*c*_ is the local characteristic impedance, that is *Z*_in_ at high frequencies which are deemed to be reflection-less [38]).

We applied the so-called *PQ*-method to compute *Z*_*c*_ from simultaneous *P* and *Q* signals, and the *PU*-loop method to directly estimate local wave velocity, *c*, from *P* and flow velocity, *U* (i.e. *Q*/*A*, where *A* is the vessel cross-section area) signals [29,36–38]. These two methods were preferred to the usual definition of $c=\sqrt{A/\rho \;dP/dA}$ because of their robustness, flexibility and independence of the model-specific *P*–*A* relationship. The *PQ*-loop and *PU*-loop methods are illustrated in figure 2*d*,*e*, where *Z*_*c*_ and *ρc* (*ρ* is blood density) are obtained as the slope of the corresponding linear tracts of the associated loop, being almost free from wave reflections.

Beside the assessment of arterial stiffness by observing local wave speed *c* [13,21,22], WA was conducted focusing on additional metrics: the reflection magnitude RM and the reflection index RI (both evaluating the amount of wave reflection with respect to forward travelling waves amplitude at a given location) [13,39], the augmentation index AI (giving a measure of the influence of the reflected wave onto the forward travelling wave resulting in the total local pressure wave) [13,29] and the pressure reflection coefficient *R*_*p*_ determined by arterial bifurcations, vessels tapering and peripheral resistance as the corresponding jump in characteristic impedance *Z*_*c*_ [6,13,29,36,40]. Such parameters are defined as

2.3 $$\mathrm{RM}=\frac{{\mathrm{PP}}_{b}}{{\mathrm{PP}}_{f}},$$

2.4 $$\mathrm{RI}=\frac{{\mathrm{PP}}_{b}}{{\mathrm{PP}}_{f}+{\mathrm{PP}}_{b}},$$

2.5 $$\mathrm{AI}=\frac{\mathrm{AP}}{\mathrm{PP}},$$

2.6 $$R_{p}=\frac{Z_{c,d}-Z_{c,p}}{Z_{c,d}+Z_{c,p}}\;\text{(at bifurcations)},$$

2.7 $$R_{p}=\sum_{e=1}^{N_{v}}\frac{Z_{c,e+1}-Z_{c,e}}{Z_{c,e+1}+Z_{c,e}}\;\text{(due to tapering) }$$

2.8 $$\mathrm{and}\;R_{p}=\frac{R_{\mathrm{down}}-Z_{c,\mathrm{out}}}{R_{\mathrm{down}}+Z_{c,\mathrm{out}}}\;\text{(at one-dimensional terminal branches)}.$$

In equations (2.3) and (2.4), PP_*b*_ and PP_*f*_ are the backward and forward pulse pressures, respectively. In equation (2.5), PP is the pulse pressure of the total (forward + backward) pressure signal and AP is the augmentation pressure [13,29], that is the difference between the systolic pressure and the pressure at the inflection point, taken as positive if the inflection point precedes systole and negative otherwise. In equations (2.6)–(2.8), subscripts *d* and *p* refer to the daughters and parent vessels of a bifurcation, respectively, where *Z*_*c*,*d*_ is computed according to the actual daughter vessels combination (e.g. vessels in parallel). Subscripts *e* = 1, …, *N*_*v*_ refer to the discretized segments of vessel *v*, *R*_down_ is the resistance downstream of the considered one-dimensional terminal branch (figure 2*f*), and *Z*_*c*,out_ is the characteristic impedance of the corresponding one-dimensional terminal artery outlet.

Parameters RM and RI were computed for each arterial vessel *v* by using mean PP_*f*_ and PP_*b*_ values over all vessel segments *e* (figure 2), while AI was computed at a single point located in the middle of the ascending aorta (no. 1) and the interosseous (no. 24) artery.

### 2.3. Wave intensity analysis

Wave intensity (WI) is defined as the power per unit vessel area carried by successive ‘wavefronts’ (infinitesimal waves) d*P*, d*U* and d*Q* which cumulatively form the original time signals *P*, *U* and *Q*, respectively, following [8,12,29,32,37]. The expressions adopted here for the forward (WI_*f*_) and backward (WI_*b*_) wave intensity components read

2.9 $${\mathrm{WI}}_{\;f,b}=\pm \frac{1}{4\rho c}{(\frac{dP}{dt}\pm \rho c\frac{dU}{dt})}^{2},$$

taking the sign ‘+’ for WI_*f*_, whereas the sign ‘−’ for WI_*b*_. Electronic supplementary material, figure S1 shows an example of WI profile computed at the aortic entrance for the supine posture, including the identification of the different forward/backward compression and decompression waves associated with WI peaks.

### 2.4. Wave trapping and moving horizon

Following the work of Davies *et al.* [14], at different sites along the arterial tree we evaluated the elapsed time between the first forward compression wave (FCW) passage and backward waves return (referred to as time of reflection, ToR). Thus, given the average speed of waves travelling downstream of the considered site (${\bar{c}}_{\mathrm{down}}$), we computed the site-specific distal point of the aorta at which reflection takes place (denoted as point of reflection, PoR), which is supposed to move downward as one proceeds along the aorta. This downward displacement suggests the definition of a moving horizon beyond which the reflected waves can no more reach the upstream site of investigation because of backward wave trapping [14–16].

**Figure 3.**
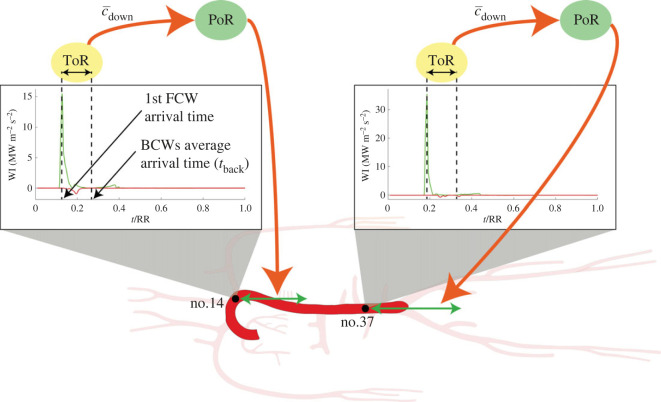
Illustration of the time of reflection (ToR) and point of reflection (PoR) computation at two different sites along the aorta. First FCW refers to the first forward compression wave corresponding to the first peak of forward wave intensity (WI), while BCWs *t*_back_ denotes the average arrival time of backward compression waves (peaks of backward WI). Time *t* is normalized with the heartbeat duration, RR, while ${\bar{c}}_{\mathrm{down}}$ is the mean downstream wave speed.

As illustrated in figure 3, computed waves ToR is the difference between the average arrival time of backward waves (*t*_back_) and the arrival time of the first FCW, where *t*_back_ is computed as

2.10 $$t_{\mathrm{back}}=\frac{\int_{\mathrm{RR}}t\;{\mathrm{WI}}_{b}\;dt}{\int_{\mathrm{RR}}\;{\mathrm{WI}}_{b}\;dt},$$

and where RR is the heartbeat duration. Then, the PoR is computed as

2.11 $$\mathrm{PoR}=\frac{\mathrm{ToR}\cdot {\bar{c}}_{\mathrm{down}}}{2},$$

where ${\bar{c}}_{\mathrm{down}}$ is the average wave speed downstream of each considered site, computed as the average wave speed over the aortic sites downstream of the current one, up to the iliac bifurcation (no. 41).

## 3. Results

Passive orthostatic stress experienced when tilting from the horizontal supine to the upright standing position triggers a number of cardiovascular responses [1,2]. In table 1, the response of the main haemodynamics parameters to posture change from $0^{\circ }$ to $90^{\circ }$ head-up is recalled (see [26] for more details). In the same table, we also specified which parameters are directly controlled by each short-term mechanism (baroreflex, cardiopulmonary reflex and cerebral autoregulation).

**Table 1.** Main haemodynamic parameters variation upon passive head-up tilt from $0^{\circ }$ to $90^{\circ }$ head-up (refer to Fois *et al.* [26,27]). cMAP and bMAP denote central (aortic) and brachial mean arterial pressure respectively, CVP is central venous pressure, HR is heart rate, SV and CO are stroke volume and cardiac output, respectively, *V*_cp_ and *V*_lb_ indicate cardiopulmonary and lower body blood volume, TPR is total peripheral resistance, *E*_ch_ are cardiac chambers elastances, *V*_*v*_ and *C*_*v*_ denote venous/venula volume and compliance, whereas *R*_*c*_ and *C*_*c*_ are cerebral arteriolar resistances and compliance. Percentage values are referred to the supine-to-standing relative parameters variation. For the parameters directly controlled by short-term regulation mechanisms, the corresponding control is indicated in the third and sixth column, where BR stands for baroreflex control, CP is cardiopulmonary control, whereas CA is cerebral autoregulation. When no specific short-term control can be identified for a given parameter, the symbol ‘—’ applies.

parameter	variation	control	parameter	variation	control
cMAP	+5%	BR	*V*_lb_	+43%	—
bMAP	+11%	—	TPR	+39%	BR + CP
CVP	−90%	CP	*E*_ch_	+6%	BR
HR	+23%	BR + CP	*V*_*v*_	−25%	BR + CP
SV	−32%	—	*C*_*v*_	−25%	BR + CP
CO	−15%	—	*R*_*c*_	−12%	CA + BR + CP
*V*_cp_	−28%	—	*C*_*c*_	+65%	CA

In the present study, the CVS model was used to study posture-induced arterial pressure and flow wave patterns, and to investigate whether and how the supine favourable and optimized (from a wave dynamics perspective) CVS configuration is maintained when approaching the standing posture. We used wave separation to explore the propagation properties of forward travelling waves throughout the arterial tree, assessing whether the optimal vessels area and impedance matching at vessel bifurcations is lost upon orthostatic stress. Then, we performed WA to quantify wave reflections by means of different indices—such as RM, RI, AI and *R*_*p*_—to understand if the production of backward waves at points of wave reflection (vessel bifurcations, tapering and terminal branches) is exacerbated by posture changing to standing. Finally, we performed WIA to investigate additional wave patterns at several sites of the arterial tree prior and after posture change, and to assess the posture-induced response of the wave trapping mechanism causing the moving horizon effect observed along the aorta.

### 3.1. Forward/backward travelling waves

**Figure 4.**
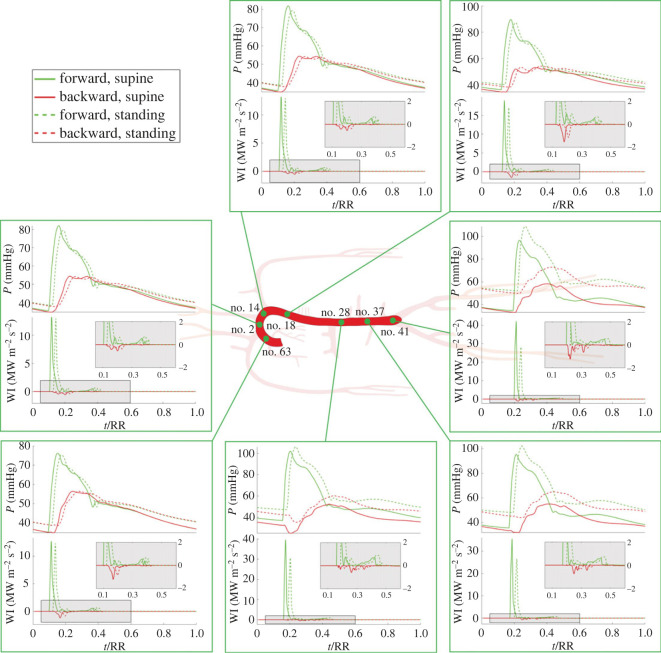
Forward (green) and backward (red) pressure (*P*) and wave intensity (WI) profiles—on top and bottom side of panels, respectively—obtained at different sites along the aorta for the supine (solid lines) and standing (dashed lines) positions. Grey boxes report a magnification of the WI profiles. Numbers associated with each aortic site correspond to the arterial tree topology illustrated in figure 1. Time *t* is normalized with the heartbeat duration, RR.

Figure 1*f*,*g* and top diagrams on all panels of figure 4 show how the separated components of pressure and flow waveforms are altered by orthostatic stress. Forward and backward pressure waves exhibit the same behaviour observed for the total (forward + backward) pressure signals after passive tilting to the standing posture [26], with both pressure components reduced in amplitude following global pulse pressure contraction (figure 1*f*,*g*). Moving along the aortic-wise direction, figure 4 shows that both forward and backward pressure signals are progressively shifted upward to higher mean pressure levels because of the increasing hydrostatic contribution encountered while moving below the heart level: standing mean forward pressure +1.6 mmHg and mean backward pressure +2 mmHg at ascending aorta no. 63 versus standing mean forward pressure +15 mmHg and mean backward pressure +15 mmHg at the iliac bifurcation no. 41 (data compared with supine). In addition, both time-normalized (with respect to the heartbeat duration RR) pressure and flow forward and backward signals are shifted in phase upon upright posture assumption—compared with supine waveforms—though almost to the same extent throughout the entire aorta (figure 4). The reason for this phase shifting lies in the raised heart rate with respect to the supine state (see table 1), altering the systolic–diastolic balance over the single heartbeat. Indeed, the higher heart rate encountered at standing posture leads to an increased systolic duration in contrast to a shortened diastole, resulting in the observed phase shifting of pressure and flow signals [26].

### 3.2. Wave intensity patterns

Figure 4 reports also forward and backward WI profiles computed at several aortic sites. Following the aortic-wise direction, we found that in the ascending aorta (site no. 63), the first forward WI peak (i.e. the first FCW) is almost identical in magnitude between supine and standing position (see also electronic supplementary material). On the other hand, the first evident backward WI peak (first BCW) detected in the ascending aorta (no. 63) is slightly reduced at standing compared with the supine state, whereas the remaining forward and backward waves do not show large variations following upright posture assumption.

Proceeding downward in the aorta, through the aortic arch (no. 2), the thoracic tracts (nos. 14 and 18) and the abdominal tracts (nos. 28 and 37) up to the iliac bifurcation (no. 41), the first FCW moves forward in time at both supine and standing positions, arriving later from the originating aortic valve connected to the left ventricle. The first FCW appears as progressively increased from upper to lower aorta (at fixed posture) due to anatomical vessels narrowing. Conversely, the first FCW peak is markedly reduced passing from the supine to the standing posture (at a fixed aortic site) because of the increased local cross-section area due to blood shift and the hydrostatic pressure contribution below the heart level.

Although forward WI profiles are qualitatively similar among sites and postures, backward WI profiles change profoundly from one site to another due to the different backward travelling waves running up the arterial tree. Indeed, the observed backward WI time profile depends on downstream wave trapping of backward waves: only reflected waves which are produced at points close enough to the considered site can be observed within the local WI time profile.

### 3.3. Wave speed and reflection indexes

Table 2 reports local wave speed *c* throughout the vessels of the one-dimensional arterial network, for the supine and the standing case. It can be seen that wave speed is progressively increased while moving along the aortic-wise direction—at fixed posture—from the aortic root (no. 1) down to the iliac bifurcation (no. 41).

**Table 2.** Standing versus supine wave velocity (*c*) computed along the arterial tree at sites specified by the numbers in the left column (refer to figure 1 and [26]). *c* values are reported as mean ± s.d. computed over the segments composing each vessel, according to the procedure illustrated in figure 2 (Δ% indicates the percentage difference between standing and supine values, *p*-values are computed via Wilcoxon’s non-parametric test for paired samples).

vessel	*c* (m s^−1^)			
	supine	standing	Δ%	*p*-value
*aorta*				
ascending no. 63	3.89 ± 0.14	4.10 ± 0.14	+5.3%	0.031
arch no. 14	4.24 ± 0.22	4.45 ± 0.22	+5.0%	0.008
thoracic no. 18	4.32 ± 0.14	4.59 ± 0.11	+6.1%	0.002
abdominal no. 39	4.85 ± 0.15	5.64 ± 0.15	+16.4%	0.002
iliac bif. no. 41	5.06 ± 0.07	6.32 ± 0.09	+24.9%	0.125
*carotid*				
external carotid no. 13	6.77 ± 0.15	6.55 ± 0.12	−3.2%	0.031
*legs*				
tibial no. 48	7.42 ± 0.11	10.78 ± 0.38	+45.2%	0.002

An increase in arterial vessels local wave speed is also observed at each considered site when passing from the supine posture to the upright standing posture, as a consequence of arterial vessels adaptation (table 1) promoted to fight orthostatic stress [3–5]. The posture-induced *c* increase is statistically significant (by Wilcoxon’s test) at all considered sites apart from the iliac bifurcation (no. 41, *p*-value = 0.125), probably due to its limited number of discretized vessel segments. The largest percentage increase in local *c* was found within leg arteries, with standing wave speed almost +45% higher than supine, whereas at carotids level we detected a slight decrease in local wave speed (−3%), potentially caused by the cerebral arteriolar vasodilation stimulated by cerebral autoregulation. Results referred to additional sites are reported in the electronic supplementary material.

Quantitative WA was carried out in terms of forward and backward pulse pressure and related indices of wave reflection. Arterial local PP_*f*_, PP_*b*_ and RM results for the supine and standing postures are presented in table 3 (RI results are reported in the electronic supplementary material). Interestingly, while PP_*f*_ is decreased to almost the same extent throughout the entire arterial tree (apart from the tibial artery no. 48) going from supine to upright standing (about −8%/−10% with respect to the supine values), PP_*b*_ shows a much wider range of variation. Indeed, PP_*b*_ shows reductions similar to those observed for PP_*f*_ only in the thoracic–abdominal aortic tracts, whereas it drops much deeper within the ascending aorta-aortic arch (−17%), carotid arteries (−31%) and terminal tibial arteries (−20%).

**Table 3.** Standing versus supine forward pulse pressure (PP_*f*_), backward pulse pressure (PP_*b*_) and reflection magnitude (RM) computed along the arterial tree at sites specified by the numbers in the left column (refer to figure 1 and [26]). PP_*f*_ and PP_*b*_ are mean values computed over each vessel as illustrated in figure 2 (Δ% indicates the percentage difference between standing and supine values).

vessel	PP_*f*_ (mmHg)			PP_*b*_ (mmHg)			RM		
	supine	standing	Δ%	supine	standing	Δ%	supine	standing	Δ%
*aorta*									
no. 1	40	37	−9.9%	22	18	−17.3%	0.54	0.49	−8.2%
no. 63	41	37	−10.4%	22	18	−17.1%	0.53	0.49	−7.5%
no. 2	47	41	−11.9%	20	16	−16.1%	0.42	0.40	−4.9%
no. 18	53	47	−11.9%	19	16	−14.8%	0.35	0.34	−3.4%
no. 37	60	54	−9.6%	23	21	−9.3%	0.39	0.39	+0.3%
no. 41	62	57	−8.7%	25	23	−8.3%	0.41	0.41	+0.5%
*carotid*									
no. 13	61	57	−6.3%	24	17	−31%	0.40	0.29	−26.4%
*legs*									
no. 48	79	85	+8.7%	34	27	−20.9%	0.44	0.32	−27.2%

As a result, RM shows significant reductions (−3%/−8%) going from supine to upright standing posture for the central aortic (ascending aorta, arch of aorta and initial thoracic aorta) regions, in line with findings of other researchers [14,17,41] (Davis *et al.* [42] found that aortic RM dropped from 0.55 ± 0.05 to 0.48 ± 0.05 going from supine to standing). We found that RM eventually recovers up to almost null variation towards the thoracic–abdominal aortic tracts and the iliac bifurcation. Besides, RM falls markedly also at carotid (−26%) and tibial (−27%) level, with the latter being caused also by the +8.7% increase in PP_*f*_. Results referred to additional sites are reported in the electronic supplementary material.

Table 4 shows the computed values for the augmentation index AI prior to and after tilting from supine to upright standing, at central (aorta) and peripheral (finger) level. AI is often taken as a proxy of arterial stiffness, although very difficult to interpret due to its strongly nonlinear behaviour [22]. It can be noted that both central and peripheral AIs drop when undergoing passive orthostatic stress, as also observed in other studies [17–20,42] (Davis *et al.* [42] registered aortic AI falling from 18%±11% to 1%±11% changing from supine to standing posture). The drop in AI should also be considered in light of the observations advanced by Wilkinson *et al.* [43], who found that elevated heart rate elicits linear falling of AI regardless of the body posture.

**Table 4.** Standing versus supine central (aortic) and peripheral (finger) augmentation index (AI). Numbers in the left column indicate the vessel where AI is computed (refer to figure 1 and [26]; Δ% indicates the percentage difference between standing and supine values).

site	supine	standing	Δ%
central AI (no. 1)	0.22	0.13	−40%
peripheral AI (no. 24)	−0.40	−0.48	−21%

### 3.4. Mechanisms of wave reflection and pressure reflection coefficients

The increase in arterial wave speed revealed at standing posture—resulting from the augmented arterial stiffness—together with the enhanced total peripheral resistance (TPR) and left ventricular activity (with respect to the supine state, see table 1), should lead to a concomitant rise of arterial wave reflections and therefore of the associated indices of wave reflection [17,18,42]. To explain the paradoxical results here obtained—and reported in the literature—we analysed the main mechanisms involved in wave reflection generation, quantifying their role in giving rise to reflected waves by means of the pressure reflection coefficient, *R*_*p*_. As the main sources of arterial wave reflections associated with *Z*_*c*_ mismatch, we included: (i) arterial bifurcations, (ii) arterial tapering, and (iii) peripheral resistance. Significance of *R*_*p*_ distributions over arterial vessels between postures was tested via Wilcoxon’s non-parametric test for paired samples.

**Figure 5.**
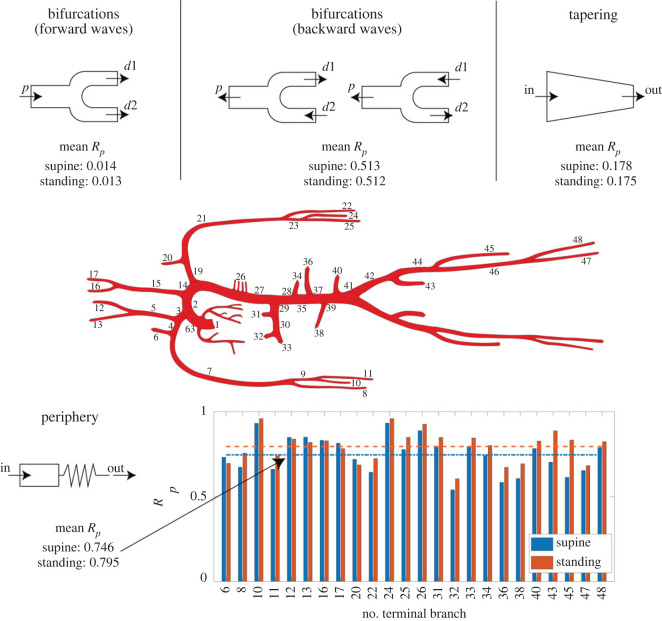
Pressure reflection coefficient, *R*_*p*_, at supine and standing postures. Left and central panels on top: mean *R*_*p*_ values at arterial bifurcations for forward (left) and backward (centre) wave transmission (*p*: parent vessel, *d*1: first daughter vessel, *d*2: second daughter vessel); right panel on top: mean *R*_*p*_ values due to vessels tapering (in: inlet section, out: outlet section); on bottom: *R*_*p*_ values due to peripheral resistance (mean values on the bottom-left side, arterial vessels are indicated by numbers with reference to figure 1).

(i) Arterial vessels are typically well matched at bifurcations in the forward direction in terms of cross-section area, though they are not in the backward direction. This means that forward wave reflections are minimized at arterial bifurcations, conversely backward wave propagation is strongly discouraged [14,29]. We found that the vessels area matching at bifurcations (in the forward direction) does not show much variation upon tilting from supine to upright standing, albeit statistically significant. We found that pre-tilt arterial tree-averaged (*A*_*d*_/*A*_*p*_)_mean_ = 1.193 (*d*: daughter vessels, *p*: parent vessel, single-bifurcation *A*_*d*_/*A*_*p*_ computed averaging the corresponding time-varying parent and daughter vessels cross-section area over a single heartbeat) versus post-tilt (*A*_*d*_/*A*_*p*_)_mean_ = 1.187 (*p*-value < 0.001). Both results are still close to the theoretical optimum value predicted by Murray’s law: (*A*_*d*_/*A*_*p*_)_opt_ ≃ 1.25 [13] (full data reported in the electronic supplementary material).
  Going from supine to standing, both forward and backward wave transmission at bifurcations show non-significant variation in terms of *R*_*p*_ mean values (figure 5, top-left and top-centre), with forward supine (*R*_*p*_)_mean_ = 0.0140 versus forward standing (*R*_*p*_)_mean_ = 0.0135 (*p*-value = 0.56), and backward supine (*R*_*p*_)_mean_ = 0.5133 versus backward standing (*R*_*p*_)_mean_ = 0.5124 (*p*-value = 0.64). As anticipated, forward waves show very low mean *R*_*p*_ value throughout the arterial tree, whereas backward waves are in all cases much worse transmitted with respect to forward propagation, with higher mean *R*_*p*_ values (full diagrams reported in the electronic supplementary material).
(ii) Arterial tapering is thought to play a key role in generating wave reflections [29–31], albeit its quantification in terms of *R*_*p*_ has not been clarified yet. As it can be seen in figure 5, top-right side, tapering produces a much larger amount of forward waves reflection compared with bifurcations. Again, going from supine to upright standing, mean *R*_*p*_ due to arterial tapering does not change significantly, with supine (*R*_*p*_)_mean_ = 0.178 versus standing (*R*_*p*_)_mean_ = 0.175 (*p*-value = 0.14). Full diagrams are reported in the electronic supplementary material.
(iii) Peripheral resistance is widely believed to produce the largest amount of forward wave reflections [6,13,29]. In the bottom panel of figure 5, we report values of *R*_*p*_ at peripheral terminal arterial branches for the supine and standing case. The increased TPR following assumption of the standing posture leads to higher values of post-tilt *R*_*p*_, with supine (*R*_*p*_)_mean_ = 0.746 versus standing (*R*_*p*_)_mean_ = 0.795 (*p*-value < 0.001).

Figure 5 shows that neither arterial bifurcations (both in the forward and backward directions) nor vessels tapering vary significantly—in terms of *R*_*p*_—changing from supine to standing posture. Therefore, such mechanisms do not cause the observed reduction in wave reflection detected at central level. Given the steep TPR rise upon upright tilting, wave reflection at peripheral sites shows the largest variation in terms of *R*_*p*_ with respect to the supine levels. Figure 5 highlights that *R*_*p*_ at peripheral sites grows markedly at almost all arterial terminal branches, apart from at external/internal carotids and vertebral arteries (nos. 6, 12, 13, 16, 17, 20) connected with the cerebral circulation. These vessels exhibit a decrease in *R*_*p*_ going from supine to upright standing, and interestingly they are the only vessels showing an increase in *A*_*d*_/*A*_*p*_ (electronic supplementary material) and a decrease in local wave speed *c* (table 2) upon tilting to upright position. The reason for this different behaviour of cerebral arteries—compared with the remainder of the arterial tree—is linked to the cerebral autoregulation mechanisms acting such that cerebral vessels tend to vasodilate in response to a blood migration from the upper to the lower body regions (see table 1), in order to preserve stable levels of cerebral perfusion. Indeed, by observing the computed RM values at terminal arteries (table 3), PP_*b*_ is strongly reduced at terminal carotid arteries (e.g. −31% at right external carotid no. 13) upon assuming the upright posture. This reduction leads to the −26% decrease registered for upright standing RM at right external carotid artery. Such strong decrease of wave reflection occurs at each of the six cerebral arteries' terminal branches, giving rise to weakened backward waves which propagate back to the arch of aorta, probably explaining the reduced reflection indices found at central level. On the contrary, backward propagation of waves originated at tibial level (this latter showing a deep fall in RM as well, going from supine to standing) cannot reach the iliac and abdominal aorta because of backward wave trapping resulting in strong damping of backward propagating information, as evidenced by the negligible RM variation registered between supine and standing in the abdominal aorta (table 3, sites nos. 37, 41).

### 3.5. Wave trapping and moving horizon

Some authors have addressed the problem of wave trapping across the arterial tree [14–16]. It is currently believed that, due to the scarce impedance matching at bifurcations for backward propagating waves, reflected waves originated at each reflection site cannot travel up the entire arterial tree reaching the aortic root, due to re-reflection occurring especially at bifurcations.

Davies *et al.* [14] suggested the existence of a ‘horizon effect’ along the human aorta. This effect explains why backward-reflected waves seem not to come from a distinct point of reflection located at the iliac bifurcation—as it was widely believed—but rather from multiple reflection sites distributed along the aortic length. Because of backward wave trapping, at a given upstream site only backward waves reflected from downstream points which are close enough are visible, as illustrated in figure 3 and highlighted by backward WI profiles shown in figure 4. In these plots taken at successive sites along the aorta, it is evident how the first BCWs (red peaks) seem to move downward as we proceed along the aorta, pushing the associated reflection site forward and behaving like a moving horizon of wave reflections (figure 3 shows the moving horizon displacement in the downstream direction).

**Figure 6.**
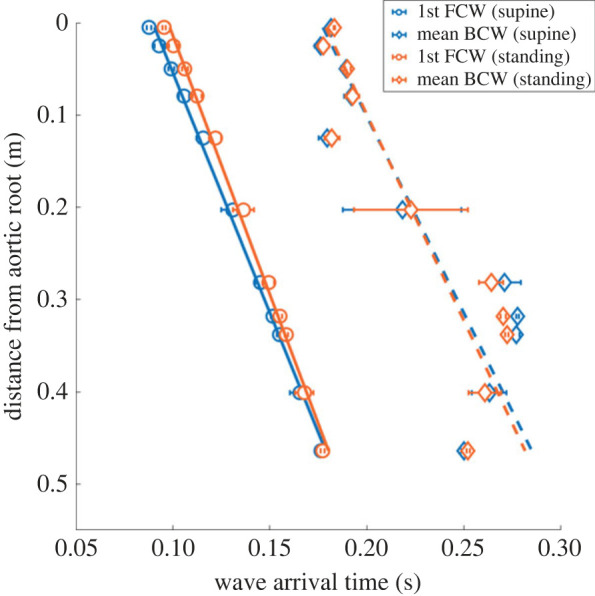
First FCW arrival times (circles and solid interpolating lines) compared with average BCW arrival times (diamonds and dashed interpolating lines) for the supine (blue) and standing (orange) position, computed at each aortic location.

In figure 6, we report—for each site and posture—the arrival time of the first FCW (circles and solid interpolating lines) and the average arrival time of backward waves (from equation (2.10), diamonds and dashed interpolating lines). From these values we computed the elapsed time of reflection, ToR, employed to estimate the distal point of reflection, PoR, of travelling waves along the aorta, reported in table 5. For the supine position, we obtained results in very good agreement with Davies *et al.* [14]. Furthermore, we found that tilting to standing position does not alter the ToR to a significant extent, while standing PoR are shifted downward with respect to the supine values by about +5% at the aortic root, and up to about +27% at the iliac bifurcation. This different shifting is directly connected to the different increase of ${\bar{c}}_{\mathrm{down}}$—which rises by about +12% at the aortic root, and by almost +25% at the iliac bifurcation due to upright tilting—though not in a linear fashion. However, these results proved how backward wave trapping is preserved after posture changes.

**Table 5.** Standing versus supine downward average wave speed (${\bar{c}}_{\mathrm{down}}$) and position of the point of reflection (PoR) computed along the aorta tree at sites specified by the numbers in the left column (refer to figure 1 and [26], Δ% indicates the percentage difference between standing and supine values).

vessel	${\bar{c}}_{\mathrm{down}}\;(m\;s^{-1})$			PoR (m)		
aorta	supine	standing	Δ%	supine	standing	Δ%
no. 1	4.53	5.09	+12.4%	0.213	0.224	+5.2%
no. 63	4.55	5.18	+13.9%	0.189	0.200	+5.8%
no. 2	4.61	5.29	+14.9%	0.208	0.222	+6.9%
no. 14	4.64	5.41	+16.8%	0.200	0.217	+8.2%
no. 18	4.69	5.54	+18.2%	0.150	0.166	+10.9%
no. 27	4.77	5.69	+19.4%	0.208	0.246	+17.9%
no. 28	4.86	5.79	+19.1%	0.305	0.332	+8.6%
no. 35	4.94	5.89	+19.1%	0.311	0.339	+8.7%
no. 37	4.97	6.00	+20.8%	0.303	0.341	+12.7%
no. 39	5.03	6.18	+22.9%	0.245	0.287	+16.9%
no. 41	5.06	6.32	+24.9%	0.186	0.236	+27.2%

ANCOVA analysis of covariance was performed to test significant differences between slopes and intercepts of interpolating lines of forward and backward waves' arrival time along the aorta. For the interpolating lines displayed in figure 6, we find statistically significant difference between FCW arrival times slope (0.1922 versus 0.1782 s m^−1^, *p*-value = 0.02) and intercept (0.0896 versus 0.0976 s, *p*-value ≃ 0) between the supine and standing posture, respectively; conversely, no significant difference is detected between average BCW arrival times slope (0.2344 versus 0.2236 s m^−1^, *p*-value = 0.84) and intercept (0.1762 versus 0.1778 s, *p*-value = 0.91) between the supine and standing posture, probably due to the larger data variability. Instead, separate comparison of supine and standing interpolating lines of FCW arrival times versus average BCW arrival times leads to no statistically significant difference between slopes (supine: 0.1922 versus 0.2344 s m^−1^, *p*-value = 0.32; standing: 0.178 versus 0.2236 s m^−1^, *p*-value = 0.22), while intercepts differ significantly (supine: 0.0897 versus 0.1762 s, *p*-value ≃ 0; standing: 0.0976 versus 0.1777 s, *p*-value ≃ 0).

## 4. Discussion and conclusion

In spite of the fact that posture-induced arterial wave dynamics were already investigated by a number of researchers [4,17–20,41,42], the mechanisms inducing observed wave pattern responses to passive assumption of the standing posture remained unclear. In the present study we employed WA tools [13,29] to shed light on the arterial wave patterns, wave intensity profiles and related wave transmission/reflection phenomena triggered after simulated passive orthostatic stress, achieved via head-up tilting.

Our model describes only acute assumption of standing posture, since longer time-scale mechanisms regulating blood pressure and volume over long-duration passive standing are not accounted for in our modelling framework. We are also aware that our definition of pressure reflection coefficient to quantify tapering reflections is a first attempt to capture a key mechanism of backward waves production. In addition, a one-dimensional description of the venous circulation is lacking and would certainly represent an improvement to our study. However, one-dimensional modelling of the venous system is still poorly widespread due to the limited knowledge available concerning critical flow conditions in veins and the mechanics of venous collapse. For this reason, we preferred not to extend the one-dimensional modelling to the venous circulation at this time.

In conclusion, our work sheds light onto arterial wave dynamics following passive change of posture. We found that the CVS shows excellent conservation of wave dynamics upon orthostatic stress. In fact, in spite of all remarkable haemodynamic alterations and vascular adaptations—such as the augmented vascular stiffness, wave speed and cardiac rhythm—associated with head-up tilting, the standing posture does not lead to larger production of wave reflections, since pressure reflection coefficients associated with arterial bifurcations, tapered vessels and at peripheral arterial branches do not increase in a relevant way. All indices of wave reflection reveal a reduced reflected waves amplitude at central level (ascending aorta, aortic arch and initial thoracic aorta), probably because of the diminished reflected waves amplitude coming from cerebral districts (due to cerebral autoregulation-induced arterial vasodilation). However, these indices show almost null variation with respect to supine values proceeding towards the lower thoracic and abdominal aorta. Thus, the supine favourable functioning of the human arterial circulation is guaranteed with posture changing to standing, as wave trapping is maintained as a protection for the heart and the aortic valve against reflected waves along with the preservation of the near-optimum area matching at bifurcations for the efficient transmission of forward waves.

## Data Availability

Additional results, supporting data, mathematical details and parameter settings of the model employed in this study are included in the electronic supplementary material [44]. Data and code are available from the Dryad Digital Repository: https://doi.org/10.5061/dryad.wpzgmsbqx [45].
